# Hospital Meetings, &c.

**Published:** 1898-11-19

**Authors:** 


### THE INSTITUTIONAL WORKSHOP.

### HOSPITAL MEETINGS, &C.

### ADDENBROOKE'S HOSPITAL, CAMBRIDGE.

A special meeting of the governors of the above institution was held on Monday, under the presidency of Mr. C. J. Clay, J.P., for the purpose of considering the report of the Special Committee on the recommendations of Sir Henry Burdett in reference to certain alterations in the constitution and the management of the hospital. Considerable interest has been displayed in Sir Henry Burdett's report, and there was a full attendance of governors.

Dr. MacAlister said it fell to him as chairman of the Special Committee, to propose the adoption of the first recommendation in the report that had been circulated among the governors. For the information of those who were not fully acquainted with the matter, Dr. MacAlister said a special committee was appointed to look into the whole question of the finances of the hospital with a view of ascertaining whether the accounts could not be balanced with more success than had been the case hitherto. After some preliminary consideration, the committee advised that an expert should be called in to give them his opinion as to the method of making the hospital a greater success. The expert called in was Sir Henry Burdett, who was known throughout the hospital world, and who had given an enormous amount of attention to the administration of hospitals in every part of the country. Sir Henry Burdett accordingly presented an elaborate report, which, by order of the court, was circulated to every governor of the hospital, by whom, he had no doubt, it had been thoroughly considered. Sir Henry recommended a number of modifications in their method of administration, and these were submitted to the committee, consisting of the original Finance Committee, with certain additions, who, after carefully considering the details, agreed upon the report which came up for consideration that day. The first recommendation was: "That a Committee of Management, consisting of eighteen governors, together with a chairman, should be appointed annually, whose duties should include those at present assigned to the Weekly Board, the Select Governors, and the Finance Committee." This meant that in the interests of the good government of the hospital the Select Governors and the Finance Committee at present appointed from year to year should be incorporated as one single board of management, and entrusted with the responsibility of the management of the hospital. It was then proposed that this larger Committee of Management should sub-divide itself into a financial sub-committee, who would hold their weekly meetings as before, and the larger Board, including both, would meet, say, monthly, or as often as was desired, as a check upon the doings of the Finance Committee, thus adapting present arrangements to present requirements.

Mr. J. Clarke seconded the proposition.

Dr. Waraker proposed that the recommendation be not received. He had hoped that the recommendations would have been taken *en bloc*. These recommendations followed upon the report of the committee, which was really the report of Sir Henry Burdett, and he thought it was supported solely because it was supposed Sir Henry Burdett was an expert on medical matters and hospital management. Sir Henry Burdett was not by any means universally regarded as such, at all events in London. At the last meeting of the governors Sir Henry Burdett urged them not to accept anything except that which was supported by precedent and justified by experience. It was singular that after having said that he should have proposed an entire subversion of their constitution, a revolutionary measure for which there was no precedent, for which there was no experience and no justification. Sir Henry Burdett proposed to uproot a constitution which had lasted for a very long time, which was supported by precedent, and justified by long experience and by success; he proposed a new-fangled scheme which had neither experience. nor precedent, nor any mortal thing to recommend it. It was, indeed, as bad and as mischievous a scheme as could possibly be devised. In the success, the almost unexampled success, the continued and uninterrupted success of that hospital, they saw strong reasons why they should not upset the administration under which those results had been effected. The fact of their success, he maintained, justified the existence of the present system, and negatived the propriety of the change that it was proposed to make. The success that had been achieved had been due to a great extent to the existing constitution. The present administration had had the effect of securing the right persons for the right places, and he ventured to think that no one of the governors would be inclined to impugn or to complain of any member of the staff. The new scheme would have the effect of disfranchising the governors, and their privileges would, in fact, be taken away. The right, for instance, of the governors to recommend patients would be taken away. That, he was convinced, would at once cause their list of subscriptions to shrink very materially, for he knew as a fact that there were persons who, if this privilege were taken away, would at once withdraw their support. He wished to speak in the most courteous terms of Sir Henry Burdett, but that did not entirely justify his omitting all remarks of a personal character. Those remarks, however, should be as moderate as he could cause them to be; but there was something he was bound to say. He would have to show that Sir Henry Burdett's schemes were visionary, that Sir Henry was not a practical man, that he was not an expert on finance or hospital reform, or even upon medical matters. Sir Henry Burdett posed as such, he knew, and he believed in himself; he was one of those men who had a happy belie in his own omniscience without knowing the facts of the case.

Dr. MacAlister: I think Sir Henry Burdett will be here presently.

Dr. Waraker remarked that he would say what he had to say when Sir Henry Burdett put in an appearance. He was quite prepared to say in his presence what he had said behind his back. He should say nothing more about Sir Henry Burdett than was necessary. Continuing, he reiterated that the Committee of Management would have the whole of the control, executive and financial. They might sell various investments, and invest in any mortal thing—in the exploration of a Klondike, or anything of that kind. It might be said that they would never think of doing that; but people did think of unsafe investments. The committee would also be able to sell out funds for paying any deficiencies that might occur. Anything more dangerous than that he could not conceive, and it was his opinion that if such a policy were pursued it would soon land them into bankruptcy. Deficiences would be created year after year, increasing in amount until they found themselves without capital at all. The hospital had been a success under the old system, and that was a strong reason why they should not make hasty changes. That success had been testified to by Sir Henry Burdett himself, and he (Dr. Waraker) was always extremely glad when he could agree with him. In conclusion, he begged to move that this recommendation be not accepted.

Dr. Besant, in seconding the motion, observed that, after consideration, he found himself opposed to nearly all the proposals in the report, because he did not think there would be any improvement upon the present system. It was always a dangerous thing to alter present conditions unless there was good reason for it. He found it difficult to believe that this committee of 18 members, including the Executive and Finance Committees, would be any the better able to manage the affairs of the hospital than the Select Governors. He regarded the existing restriction in reference to the selling out of investments as a most important safeguard, and he hoped that the governors would be careful to retain the power they now possessed, and not allow it to go into the hands of any committee.

Dr. Cooper said that if they adopted Dr. MacAlister's resolution they practically reduced the president and governors of Addenbrooke's Hospital to mere cyphers, to becoming only the recording angels of the Weekly Board. Besides this, he would point out that the powers which it was intended to give the new committee were greatly in excess of what the Act of Parliament laid down. The Act stated that the governors should meet four times a year, and at one of their meetings might depute to a commitee certain definite powers, which certainly did not include many of those it was now sought to depute to the Committee of Management. The appointment of a vice-president, too, was going beyond their Act of Parliament. In concluding, he expressed an earnest hope that the feeling of that meeting against the appointment of a Committee of Management would be so strong that the committee who had brought up these recommendations, would see the inutility of further pursuing the matter. He hoped that the Board that day would absolutely refuse with no uncertain voice the setting up of the new constitution imposed upon them by Sir Henry Burdett. He did not wish to say anything personal, but Sir Henry was not called in to advise as to a new constitution, but only on the financial condition of the hospital. He had read Sir Henry's report carefully, and it did not throw as much light upon the latter point as the late Mr. Hall used to in his very exhaustive criticism on the accounts of the hospital from quarter to quarter. However, Sir Henry Burdett had been called in, and it was the worst day's work for the hospital that this had been done. The governors could have found out for themselves all that he had told them, and he looked upon the money they had paid to him as wasted.

Sir Henry Burdett: Nothing was paid to him.

Dr. Cooper: Then I don't think that increases the value of his recommendations, and I am very glad to hear that the hospital was put to no expense in the matter.

A Governor explained that Sir Henry had been good enough to present to the hospital the honorarium which had been voted to him.

Dr. Cooper: I am very glad to hear that, for it confirms what I have said. Sir Henry evidently thought his recommendations were of no value at all.

Mr. Spalding said it was only fair to say that the committee took upon itself all responsibility for the recommendations they had made. They did not wish to throw the onus of the report upon Sir Henry Burdett's shoulders at all. He regretted that Sir Henry did not arrive earlier in order that he might have heard the opinions expressed upon the recommendations, which had been described as some of the most mischievous ever made. Previous speakers had also said that Sir Henry Burdett had little or no experience in the matter of hospitals, and that his chief recommendation was that he believed in himself. Such remarks as these, and he greatly deprecated their utterance, needed no answer. As to the recommendation they were discussing, it was, in his opinion, a remarkably good one. Over and over again, under the present *régime*, there had been illustrations of what might have happened if it had not been for the self-restraint and temperance exercised by the governors. The select governors simply acted on sufferance, and if any governor had a fad which he wished to air it was the easiest matter in the world for him, with the assistance of one or two governors who never attended except on that one occasion, to carry his fad out successfully. Dr. Cooper had stated that the great success of the management of Addenbrooke's Hospital was due to the existing constitution. But had Addenbrooke's management been a great success? If it had, why did they call in expert advice? Surely it was due to the fact that the existing management had not quite been a success that such a course had been taken.

Mr. Parker also supported the resolution. Dr. Cooper, he said, had urged that in carrying out the recommendations they would be altering the constitution and going against the Act of Parliament. The power to create such a committee, however, was well within the Act, for, after all, the Committee of Management only differed from the present Weekly Board in the fact that the former would be a fixed committee while the latter varied periodically.

Dr. Lathom could not see why the Weekly Board and the Finance Committee, which for the last 30 years had been discharging their duties most efficiently, should be replaced by a committee about which the governors knew nothing. They did not know how it would act, nor yet how it was to be appointed, and still it was to be entrusted with unlimited powers with regard to expenditure. The present Weekly Board could not spend more than £10 without appealing to the governors, and he thought it was well that there should be such a check as this on their expenditure.

Dr. Boulter expressed his deep regret that several speakers had gone out of their way to speak in derogatory terms about the gentleman who, at much expenditure of time and trouble, had given them the benefit of his advice. He regretted it the more because these personal remarks were not necessary to the working out of their case. He was in favour of the appointment of a Committee of Management, but took exception to some of the powers proposed to be conferred upon it.

A discussion then took place as to whether the carrying of the resolution bound the meeting to favour the subsequent resolutions, in the course of which Dr. MacAlister pointed out that the resolution before the Governors simply had the effect of amalgamating two committees already existing. If his resolution was carried, the court would have to define the duties of that committee by subsequent resolutions, each of which would have to come up for discussion.

Sir Henry Burdett expressed his regret that the imperfect train service to Cambridge in the morning had prevented his arrival at the commencement of the meeting, when he might have heard all that the governors could say against himself as an individual and, what was more important and interesting, what they could say against the recommendations. Whatever merit or demerit those recommendations had, they rested upon a rock, the rock of experience. He was sure they would excuse him for saying that he had done too much service for the hospitals of the world and of this country to defend himself or his reputation. His reputation rested upon his public work, and if those gentlemen who objected to him and said that he had no experience on the question had thought over the matter—or, better still, if they had done his work the justice to inquire what it in reality was—they would never have used words which he was sure they on calmer reflection must regret. The personal aspects of the question might, perhaps, be allowed to end with that. Turning, therefore, to the real business of the day, this was the present state of affairs. They had an old, great, and reputable institution which had done splendid work in the past, but which was now hampered by want of money. The governors had to consider if there was any course, based upon experience, which they could properly adopt with the hope that their financial position would improve, and in so doing would enable them to do even better work in the future than they had done in the past. It was on this line that he hoped the governors would record their votes that day. He urged them not to vote from any prejudiced idea that anyone was to be interfered with, or that it was perhaps better to leave well alone, but let them vote on the principle that in the history of institutions, as in the history of nations, a time must come when it was well to overhaul the ship and to go over the whole of the machinery, so as to make such modifications as time might have rendered necessary. Let them remember that if they came to the conclusion that it was better to do nothing, then on the gentlemen who voted for that decision rested the entire responsibility for the financial deficiency of the institution in the future. The points raised against the recommendations divided themselves under three heads: First, law; secondly, expenditure; and, thirdly, income. Taking the legal aspect first, everything which had been recommended had been done in accordance with the Act, for in order that there might be no mistake Counsel's opinion was taken in the person of Mr. Bristowe, who had given more attention to this kind of work than anyone with whom he (Sir Henry) was acquainted. The governors might take it as authoritative that the changes recommended were absolutely in accordance with the Act and legally accurate. As to the question of expenditure, one of the speakers said he thought it was a great thing that the Weekly Board had not the power to make an expenditure beyond £10 in one sum without obtaining the permission of the governors. His experience was that it would be impossible—outside Cambridge at any rate—(laughter)—to get any gentlemen to serve on a board if their hands were tied in this way, and if they were to be told, "You may be the heads of large businesses spending tens of thousands a year, but we as governors cannot trust you to spend a sum of money greater than £10." With reference to income, the hospital was in an extraordinary condition. It reflected the greatest honour and credit upon the people of Cambridge that the hospital had so large a voluntary income, for with the exception of the Ladies' Committee (which he believed was a recent inception), subscriptions had practically come to the hospital without any machinery for getting them in. It was because of the lack of this machinery, and because in spite of this lack the subscriptions had come in that he believed that if business principles were brought to bear upon getting the money for Addenbrooke's Hospital, the hospital could get all it wanted, and more too, for carrying on its work. Finally, it was essential that there should be a body, directly responsible to the governors as their servants and delegates, who should be held responsible for the administration of the institution.

Dr. MacAlister very briefly replied, and

On being put to the vote the resolution was lost, 15 voting for it and 16 against.

Dr. MacAlister and Mr. J. Clarke demanded a poll, and it was agreed that the poll should be taken on Monday, the 28th inst.

A vote of thanks to the Chairman was passed, and

The meeting terminated.

### THE BRITISH HOME FOR INCURABLES.

At the half-yearly meeting of this institution, the Chairman (Mr. F. A. Bevan) explained that the principle point of interest before the meeting was that the board had determined to carry out the erection of a large hall for entertainments for the patients, over which there would be bedrooms. The present accommodation was too limited for the number of patients. The board were desirous that the beds should be endowed. The total cost would be £14,000, and they had confident hopes that this sum would be forthcoming. He also referred to the want of stained-glass windows for the chapel. He wished special notice to be taken by those who were making their wills that it was important to write the full name of the home. The election of in-patients and pensioners then took place.

